# Causal Association and Shared Genetics Between Asthma and COVID-19

**DOI:** 10.3389/fimmu.2022.705379

**Published:** 2022-03-21

**Authors:** Ancha Baranova, Hongbao Cao, Jiu Chen, Fuquan Zhang

**Affiliations:** ^1^ School of Systems Biology, George Mason University, Fairfax, VA, United States; ^2^ Research Centre for Medical Genetics, Moscow, Russia; ^3^ Institute of Neuropsychiatry, The Affiliated Brain Hospital of Nanjing Medical University, Nanjing, China; ^4^ Department of Psychiatry, The Affiliated Brain Hospital of Nanjing Medical University, Nanjing, China

**Keywords:** asthma, COVID-19, Mendelian randomization, inflammation, OAS2, ABO, ATXN2

## Abstract

**Objectives:**

Recent studies suggest that asthma may have a protective effect on COVID-19.We aimed to investigate the causality between asthma and two COVID-19 outcomes and explore the mechanisms underlining this connection.

**Methods:**

Summary results of GWAS were used for the analyses, including asthma (88,486 cases and 447,859 controls), COVID-19 hospitalization (6,406 hospitalized COVID-19 cases and 902,088 controls), and COVID-19 infection (14,134 COVID-19 cases and 1,284,876 controls). The Mendelian randomization (MR) analysis was performed to evaluate the causal effects of asthma on the two COVID-19 outcomes. A cross-trait meta-analysis was conducted to analyze genetic variants within two loci shared by COVID-19 hospitalization and asthma.

**Results:**

Asthma is associated with decreased risk both for COVID-19 hospitalization (odds ratio (OR): 0.70, 95% confidence interval (CI): 0.70-0.99) and for COVID-19 infection (OR: 0.83, 95%CI: 0.51-0.95). Asthma and COVID-19 share two genome-wide significant genes, including *ABO* at the 9q34.2 region and *OAS2* at the 12q24.13 region. The meta-analysis revealed that *ABO* and *ATXN2* contain variants with pleiotropic effects on both COVID-19 and asthma.

**Conclusion:**

In conclusion, our results suggest that genetic liability to asthma is associated with decreased susceptibility to SARS-CoV-2 and to severe COVID-19 disease, which may be due to the protective effects of ongoing inflammation and, possibly, related compensatory responses against COVID-19 in its early stage.

## Introduction

The COVID-19 pandemic due to the severe acute respiratory syndrome coronavirus 2 (SARS-CoV-2) infections caused a public health crisis worldwide. By the end of March 2021, the COVID-19 pandemic has incurred 128 million infections worldwide, including close to three million deaths, with a mortality rate of 2.2%, according to Johns Hopkins Coronavirus Resource Center (https://coronavirus.jhu.edu/map.html). Although the majority of infected persons experience mild no obvious symptoms, approximately 10-20% of people with COVID-19 infection need hospitalization (1, 2). In hospitalized patients, comorbid hypertension, obesity, and diabetes are common (1, 2).

There is substantial variability in terms of symptoms, severity, and prognosis of the disease. Infected individuals with older age or medical complications are more likely to develop severe symptoms, with some young and seemingly healthy individuals also having serious outcomes. Host genetics is considered to play an essential role in an individual’s propensity to contract infectious diseases (3). Other medical conditions may exert an influence on an individual’s susceptibility to COVID-19 both by disease-driven conditioning of the immune system, and by shared genetic variations, which may either predispose to comorbid conditions or aid in resisting disease phenotype.

As COVID-19 disease is known to affect both upper and lower airways, it is not surprising that the links between SARS-CoV-2 severity and pre-existing lung inflammation were soon uncovered. In particular, in lung epithelium cell datasets from SARS-CoV-2 infection and idiopathic pulmonary fibrosis, transcriptomic analyses pinpointed a set of shared pathways and hub genes (4). Similarly made observations genetically connected SARS-CoV-2 withpulmonary arterial hypertension (5).

It is commonly accepted that asthmatics and patients with respiratory allergies have increased susceptibility and severity for viral infections (6). Therefore, asthma was initially considered as a risk factor for COVID-19, and a higher prevalence of asthma in COVID-19 hospitalized patients has been reported (7). Asthma was reported to be associated with a higher risk of morbidity in COVID-19 patients (8). However, some studies reported that the prevalence of asthma in patients with COVID-19 is lower than expected (9, 10), suggesting that having asthma may exert a protective effect (11). Some evidence indicates that asthma is not associated with outcomes of COVID-19 (12–14). Until now, the relationship between COVID-19 and asthma remains controversial and is under active debate (15–18). The associations reported by observational studies may suffer from limited support for causality. Therefore, there is an urgent need to determine their association using more fundamental evidence and to elucidate the mechanisms underlying the association between COVID-19 and asthma.

Mendelian randomization (MR) is an analytic framework that utilizes genetic variants as instrumental variables to test the causative association between an exposure and an outcome (19), which has been widely used in recent studies (20–22). In particular, previous MR analyses have reported causal risk factors for a severe course of COVID-19, including body mass index and smoking intensity (23–25). In this study, summary-level Genome-Wide Association Studies (GWAS) data were utilized to test for putative causal associations between asthma and two COVID-19 outcomes. Furthermore, we annotated the COVID-19 GWAS results by performing functional analyses for the discovered genes. These asthma-related findings may shed more insight into the COVID-19 pathophysiology.

## Methods

### Study Design and Participants

Two-sample MR was employed to investigate causal relationships between asthma and COVID-19 outcomes in the summary-level GWAS datasets. The asthma GWAS dataset included 88,486 cases and 447,859 controls (97.2% of the participants were of European origins) (26). Two datasets were obtained from the COVID-19 Host Genetic Initiative GWAS meta-analyses round 4 (Release Date: October 20, 2020) (27), with outcomes including either COVID-19 hospitalization (6,406 hospitalized COVID-19 cases and 902,088 controls), or COVID-19 infection (14,134 COVID-19 cases and 1,284,876 controls). COVID-19 infection reflects the overall susceptibility to the disease, whereas COVID-19 hospitalization cases represent the relative severity of the disease. In both the COVID-19 datasets, all the participants were of European origins.

### MR Analysis and Genetic Correlation Estimation

Causality was tested using inverse variance-weighted (IVW) analysis (28). To evaluate the sensitivity, we further test the causal effect using the MR-Egger regression (29) and the weighted median method (30). The intercept from the MR-Egger model was used as a measure of directional pleiotropy. All the above analyses, and the heterogeneity analysis, were conducted using TwoSampleMR v0.5.5 (31). Single-nucleotide polymorphisms (SNPs) associated with asthma at genome-wide significance (P < 5.0E-8) were selected as instrumental variants and further pruned using a clumping r^2^ cutoff of 0.01. The genetic correlations of asthma with COVID-19 outcomes were calculated using linkage disequilibrium (LD) score regression (32). Statistical significance of the analyses was accepted when P values were < 0.05.

### Annotation of the COVID-19 and Asthma GWAS Results

Functional mapping and annotation (FUMA) software was used to map SNPs to genes and identify LD-independent genomic regions (33). All genes located within 10 kb vicinity of each variant were mapped. Independent significant SNPs (IndSigSNPs) were extracted when their *P*-value were genome-wide significant (*P* ≤ 5.0E-08) and independent of each other (r^2^ < 0.6). Lead SNPs were identified as a subset of the independent significant SNPs that were in LD with each other at r^2^ < 0.1 within a 500 Kb window. Genomic risk loci were identified by merging lead SNPs located at a distance of less than 500 kb from each other. Clumping procedures were carried out in accordance with the European 1000 Genomes Project phase 3 reference panel. Due to extensive LD, the entire major histocompatibility complex (MHC) locus was merged into one region (chr6:25-35Mb). Regional association results of the loci were plotted using LocusZoom (34).

### Cross-Trait Meta-Analysis of COVID-19 Hospitalization and Asthma

We conducted a cross-trait meta-analysis to identified pleiotropic genetic variants shared by asthma and COVID-19 hospitalization. ASSET is an agnostic approach that performs cross-trait meta-analysis by allowing a subset of the input GWASs to have no effect on a given SNP (35). This technique identifies the strongest association signal by exhaustive exploration of all possible subsets of GWAS and their inputs within a fixed-effect framework.

### Tissue Specificity and Pathway Enrichment Analyses

Tissue specificity of the genome-wide genes was measured against each of the differentially expressed gene (DEG) sets from GTEx v8 (36) using the hypergeometric test (33). For each genome-wide gene, enrichment in canonical pathways was evaluated using FUMA (33). All analyses were done using R v4.0.3 or Python v3.7. A detailed description of the methods is provided in the **Supplementary File**.

### Gene Overlap Analysis for COVID-19 and Asthma

To identify overlapped risk genes between COVID-19 and asthma, we retrieved genome-wide risk genes for two traits from GWAS-catalog (https://www.ebi.ac.uk/gwas/). For COVID-19, we combined the results from GWAS-catalog and the genes identified in our present study.

## Results

### MR Analysis and Genetic Correlation Estimation

**Figure 1:**
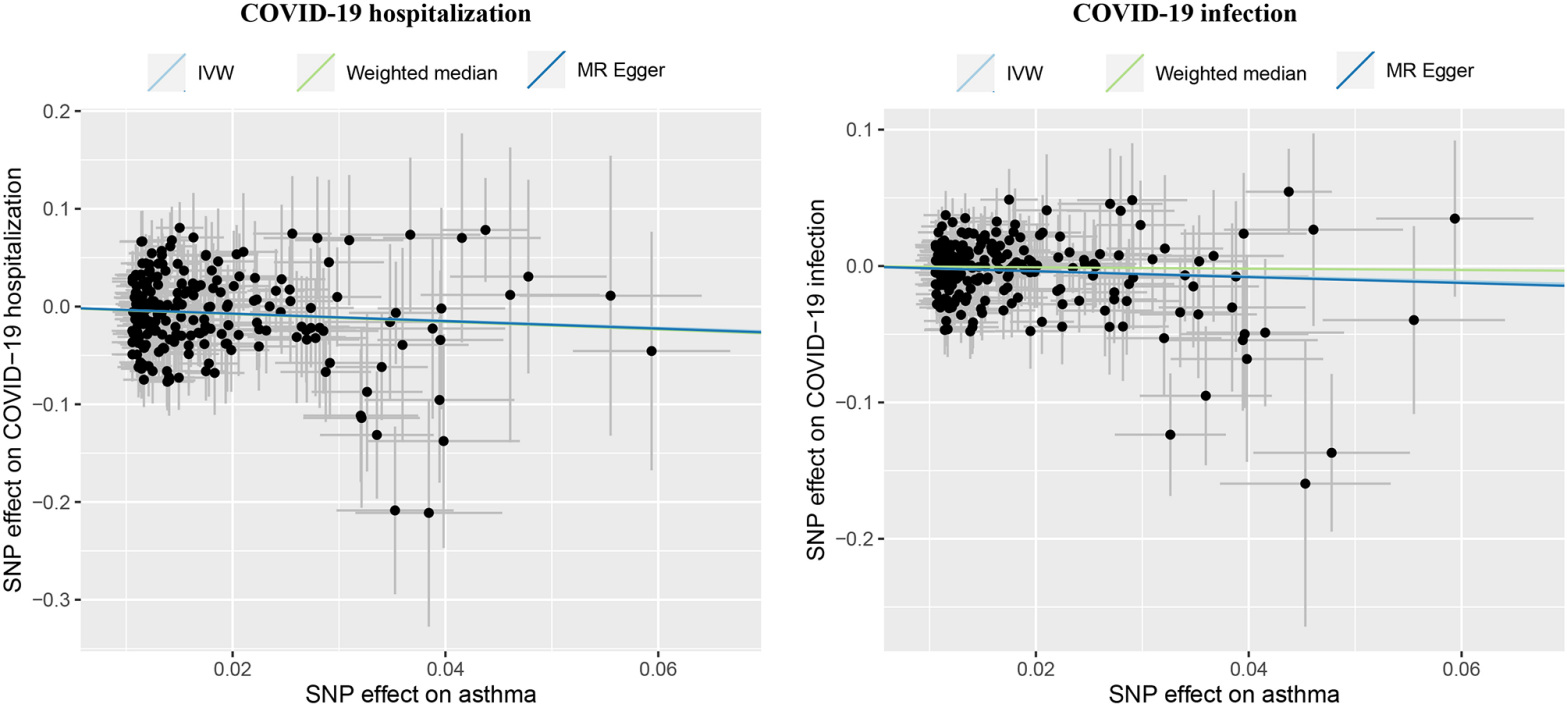
Causal effects of asthma on COVID-19 outcomes, including hospitalization and infection. IVW, inverse variance weighted; MR, Mendelian randomization. The lines denote effect sizes (b).

As shown in **Table 1** and **Figure 1**, our MR analysis unequivocally indicated that asthma is associated with decreased risk for either COVID-19 infection (OR: 0.83, 95%CI: 0.70-0.99, P = 0.037) or hospitalization (OR: 0.70, 95%CI: 0.51-0.95, P = 0.023). The sensitivity analyses suggested that the directions of causal effect estimates across the methods were the same. Tests of MR-Egger regression did not support the directional pleiotropy of the genetic instrumental variables for both the causal associations (MR-Egger intercept < 0.001, P > 0.05). The heterogeneity test did not support the existence of heterogeneity in the MR analysis (all P > 0.05). There were no genetic correlations between asthma and COVID-19 hospitalization (r = -0.03, P = 0.631) or COVID-19 infection (r = 0.11, P = 0.120).

**Table 1:** Causal effects of asthma on the COVID-19 outcomes.

Exposure	Outcome	Method	nSNP	b	se	OR [95%CI]	P	Egger_intercept	P_pleiotropy
Asthma	COVID-19 hospitalization	IVW	214	-0.359	0.158	0.70 [0.51-0.95]	0.023	3.75E-04	0.96
Asthma	COVID-19 hospitalization	Weighted median	214	-0.392	0.223	0.68 [0.44-1.05]	0.079	3.75E-04	0.96
Asthma	COVID-19 hospitalization	MR Egger	214	-0.383	0.481	0.68 [0.27-1.75]	0.427	3.75E-04	0.96
Asthma	COVID-19 infection	IVW	216	-0.186	0.089	0.83 [0.70-0.99]	0.037	4.71E-04	0.91
Asthma	COVID-19 infection	Weighted median	216	-0.048	0.131	0.95 [0.74-1.23]	0.711	4.71E-04	0.91
Asthma	COVID-19 infection	MR Egger	216	-0.215	0.27	0.81 [0.47-1.37]	0.427	4.71E-04	0.91

IVW, inverse variance weighted.

### Annotation of the COVID-19 and Asthma GWAS Results

**Figure 2:**
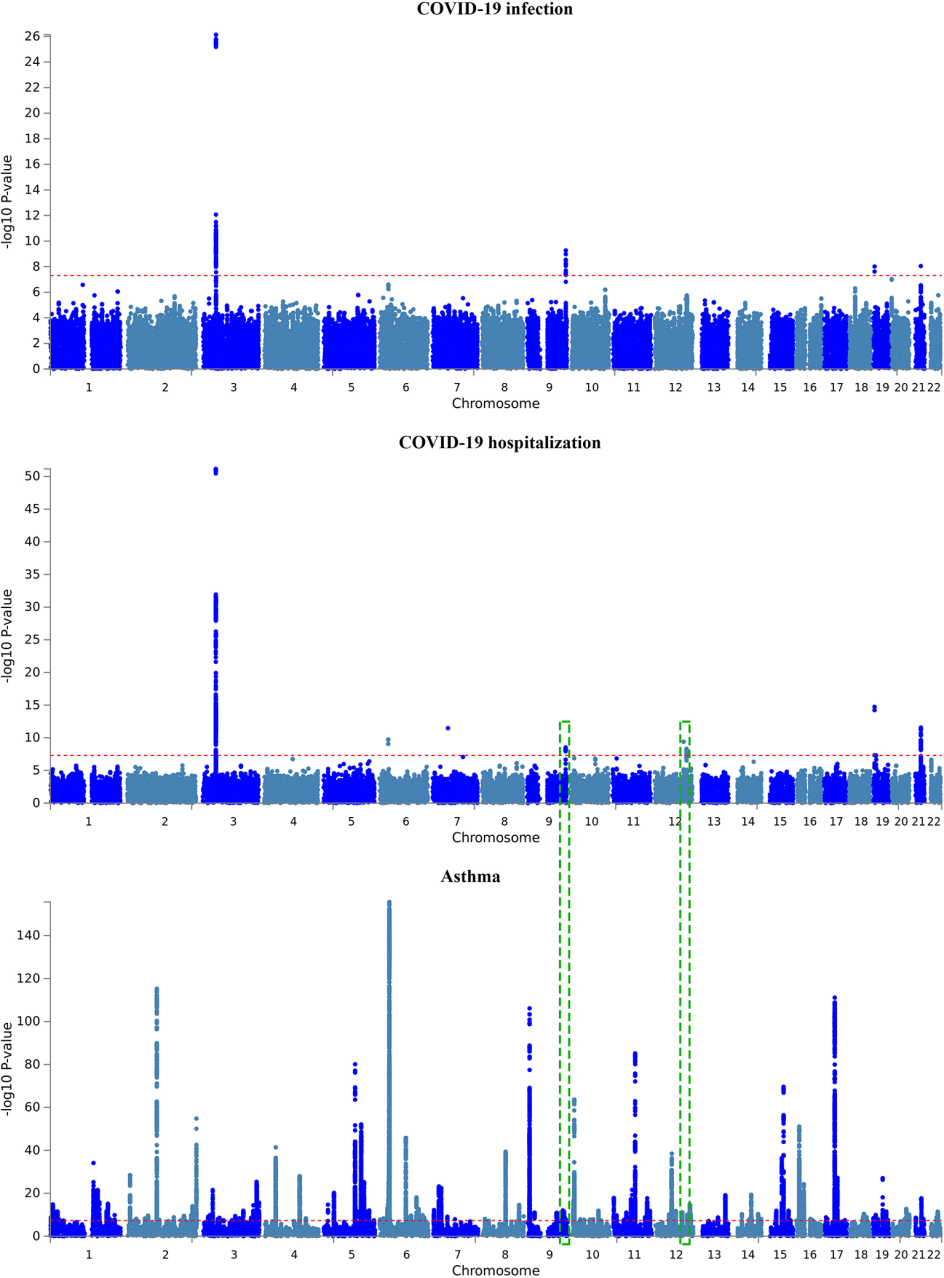
Manhattan plot of GWAS results of the COVID-19 outcomes and asthma. The x-axis is chromosomal position of SNPs and the y-axis is the significance of the SNPs (-log_10_P). Each horizontal dashed line denotes genome-wide significance level of 5E-8. Dashed green rectangles indicate the two overlapped genomic loci between COVID-19 hospitalization and asthma.

A total of six and four genomic loci were associated with COVID-19 hospitalization and with infection, respectively (**Table 2** and **Figure 2**). All the four loci implicated in COVID-19 infection overlapped with the six loci associated with COVID-19 hospitalization. For both datasets, the 3p21.31 locus had the largest amount of signals within protein-coding genes.

**Table 2:** Genomic loci influencing the COVID-19 outcomes and asthma.

Trait	Loci	SNP	CHR	Start-end	A1/A2	OR [95%CI]	P	Coding Genes
COVID-19 infection	1	rs34326463	3	45835417-46279150	A/G	1.32 [1.25-1.39]	7.37E-27	*SLC6A20;LZTFL1;CCR9;FYCO1;CXCR6;XCR1;CCR3*
COVID-19 infection	2	rs8176719	9	136132908-136149500	-/C	1.12 [1.08-1.16]	5.36E-10	*ABO*
COVID-19 infection	3	NA	19	4715016-4726931	C/A	1.10 [1.07-1.14]	9.73E-09	*DPP9*
COVID-19 infection	4	NA	21	34589235-34635053	C/G	1.10 [1.06-1.13]	9.03E-09	*IFNAR2*
COVID-19 hospitalization	1	rs35081325	3	45665765-46482683	A/T	1.82 [1.68-1.96]	6.89E-52	*LIMD1;SLC6A20;LZTFL1;CCR9;FYCO1;CXCR6;XCR1;CCR3;CCR1;CCR2;CCRL2;LTF*
COVID-19 hospitalization	2	rs622568	7	54623875-54672096	A/C	1.26 [1.18-1.34]	3.34E-12	*VSTM2A*
COVID-19 hospitalization	3	rs950088295	9	136132908-136149500	G/A	0.84 [0.79-0.89]	3.00E-09	*ABO*
COVID-19 hospitalization	4	NA	12	102990430-113444024	C/A	0.80 [0.74-0.86]	4.04E-10	*OAS1;OAS2;OAS3*
COVID-19 hospitalization	5	NA	19	4715016-4726931	C/A	1.23 [1.17-1.29]	1.85E-15	*DPP9*
COVID-19 hospitalization	6	rs13050728	21	34589235-34635053	T/C	0.83 [0.79-0.88]	2.76E-12	*IFNAR2*
Asthma	91	rs782134971	9	45835417-46279150	-/AAACTGCC	1.01 [1.01-1.02]	2.96E-08	*ABO*
Asthma	117	rs653178	12	111826477:112928596	T/C	1.01 [1.01-1.02]	1.04E-10	*SH2B3;ATXN2;BRAP;ACAD10;NAA25;TRAFD1;HECTD4;PTPN11;MAPKAPK5;TMEM116*

CHR, chromosome; BP, base position; NA, not available.

A total of 19 and 10 protein-coding genes were detected for COVID-19 hospitalization and infection, respectively (**Table 2**). All the 10 coding genes implicated in COVID-19 infection overlapped with the gene set for the COVID-19 hospitalization. Therefore, the present study revealed a total of 19 genome-wide risk genes for COVID-19, including *ABO, CCR1, CCR2, CCR3, CCR9, CCRL2, CXCR6, DPP9, FYCO1, IFNAR2, LIMD1, LTF, LZTFL1, OAS1, OAS2, OAS3, SLC6A20, VSTM2A*, and *XCR1*.

For asthma, a total of 169 genomic loci were associated with the illness. Interestingly, two loci were overlapped with those of COVID-19 hospitalization, including the 9q34.2 locus and the 12q24.13 locus (**Table 2** and **Figure 2**). The *ABO* gene within the 9q34.2 locus was implicated in both asthma and COVID-19.

### Cross-Trait Meta-Analysis of COVID-19 Hospitalization and Asthma

**Figure 3:**
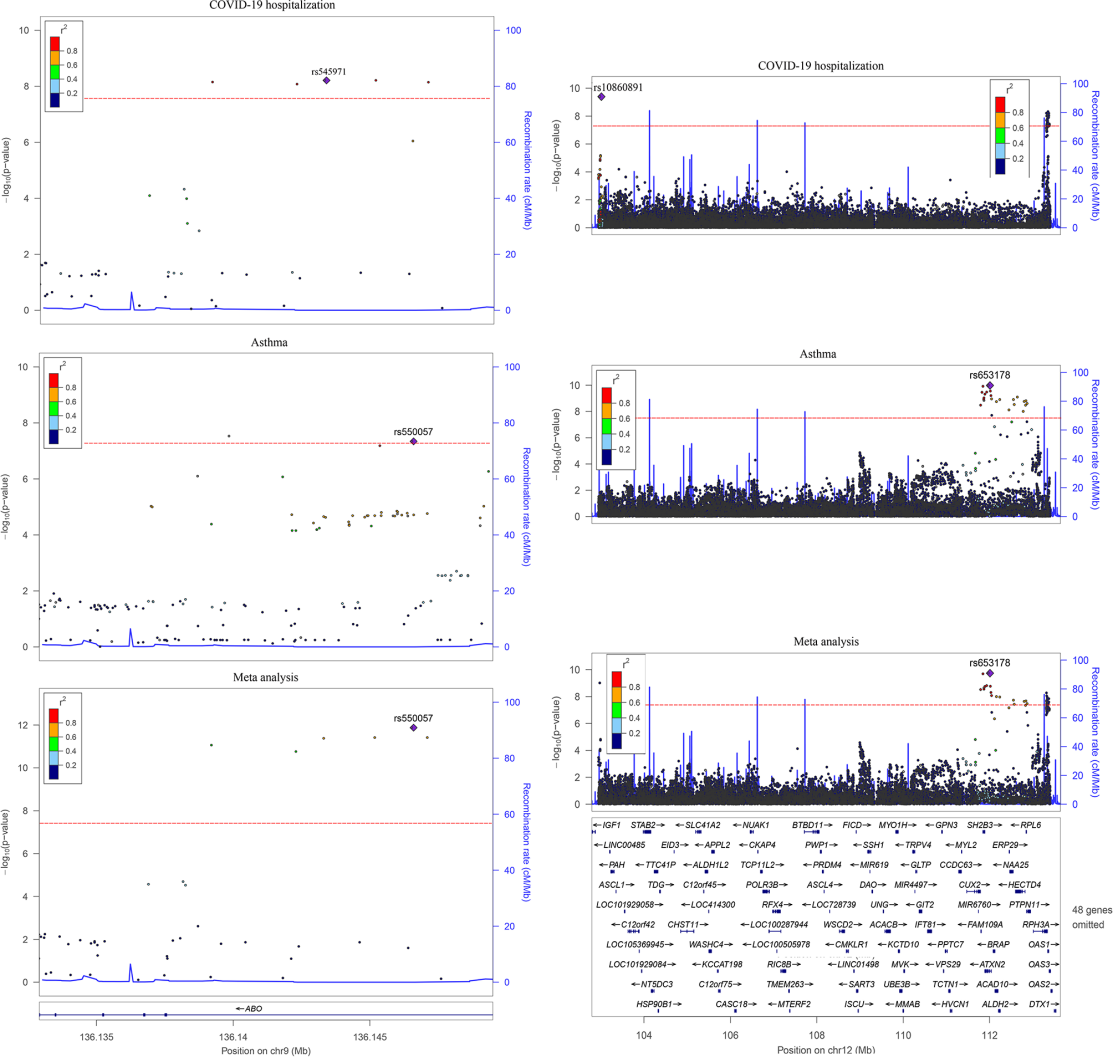
Two overlapped loci between COVID-19 hospitalization and asthma. Left is the 9q34.2 locus and right is the 12q24.13 locus in hg19. The linkage disequilibrium information is from phase 3 of the 1000 Genomes Project. The dashed line represents the threshold for genome-wide significance (P < 5.0E-08).

The cross-trait meta-analysis identified 63 significant associations (P < 5E-8), including two SNPs shared by COVID-19 and asthma (**Figure 3** and **Supplementary Table 1**). The rs1381383189 within *ABO* was implicated in both the traits in the same direction (3.00E-08), while rs35350651 within *ATXN2* was implicated in both the traits in the opposite directions (2.33E-09).

### Tissue Specificity and Pathway Enrichment Analyses

**Figure 4:**
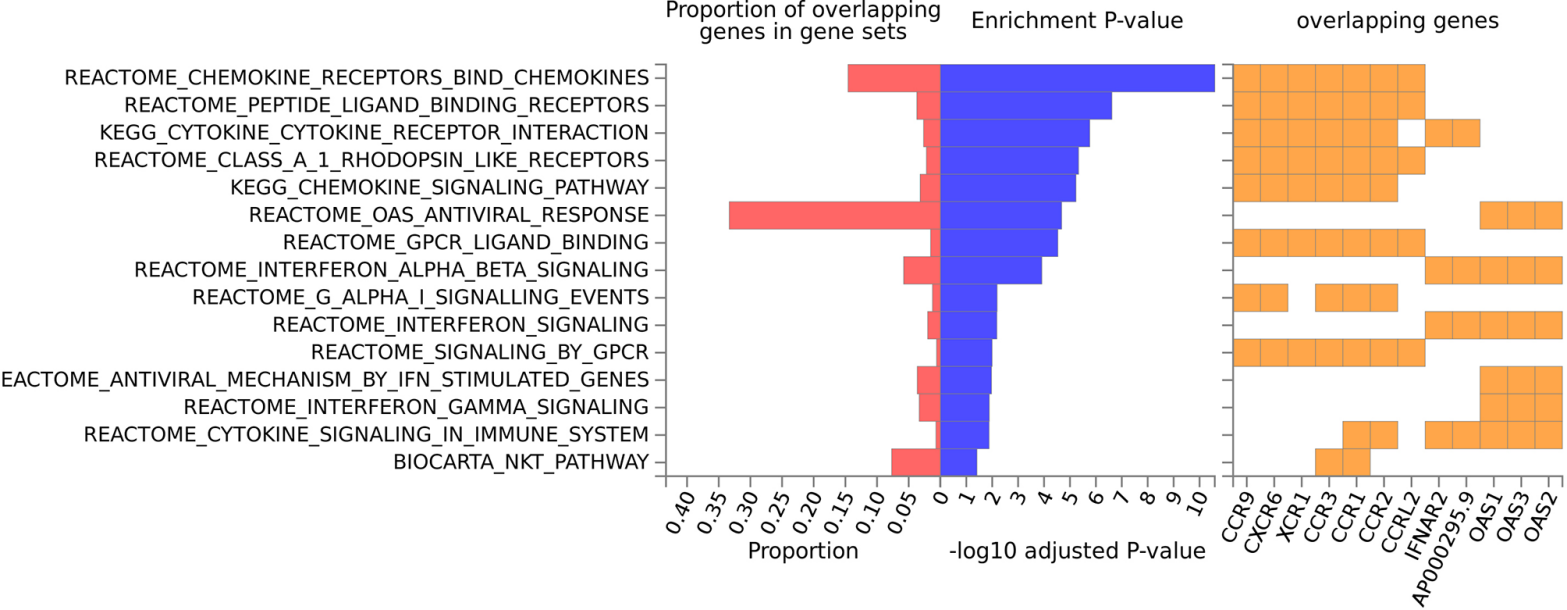
Canonical pathway analyses of the set of COVID-19 risk genes.

Gene-based tissue enrichment analysis showed that the set of 19 genes of COVID-19 was upregulated in the spleen, lung, and blood (**Supplementary Figure 1A**). SNP-based tissue enrichment analysis of asthma showed that the GWAS hits of asthma were over-represented in blood, spleen, lung, and small intestine (**Supplementary Figure 1B**). The pathway enrichment analysis highlighted multiple pathways, including cytokine interaction with their receptors, chemokine and interferon signaling, human oligoadenylate synthetase (OAS) antiviral response, G protein-coupled receptor signaling, and natural killer T pathway (**Figure 4**). These pathways are predominantly involved in the inflammatory function.

### Overlapped Genome-Wide Risk Genes Between COVID-19 and Asthma

In the GWAS catalog, there were 19 and 1,293 genes for COVID-19 and asthma, respectively. After merging with the 27 genes extracted in this study, a set of 34 risk genes for COVID-19 was formed. Overlap analysis revealed that two protein-coding genes were shared between the two conditions, namely, *ABO* and *OAS2*.

## Discussion

Our study shows that asthma has a protective effect on the risk of COVID-19 infection and hospitalization, representing a surprising departure from other common respiratory viral outbreaks. Notably, COVID-19 progression relies on the over-activation of innate immunity and ‘cytokine storms’. Predominantly allergic immune responses, which are characteristic of asthma, may mediate the protective effect of asthma against COVID-19. The receptor-binding domain of SARS-CoV-2 spike protein docks to Angiotensin-Converting Enzyme 2 (ACE2), which is encoded by the gene reported as less active in asthma patients, thus, possibly limiting the entry of the virus into the epithelium of the asthmatic’ airways (37). The expression levels of ACE2 negatively correlate with the levels of Th2 cytokines in airway epithelial cells (IL-4, IL-5, and IL-13) and with total amounts of IgE (37, 38). In asthma, the predominance of Th2 response may alleviate the viral-induced release of interferons, and downregulate the cytokine storm which is typical for advanced COVID-19, thus, preventing hospitalization.

On the other hand, both the asthma treatments, namely, inhaled corticosteroids (39), and the cross-reactivity to T cell epitopes of common airborne allergens (40) may directly decrease the risk of contracting SARS-CoV-2 infection either by alleviating inflammation or by providing pre-existing immunity.

In this paper, we identified a set of 19 protein-coding risk genes associated with COVID-19 susceptibility and severity. These genes are located within six genomic loci, with chromosome 3p21.31 displaying the peak association across the two COVID-19 datasets and encompassing a cluster of chemokine receptor genes. Thus, our study supports the 3p21.31 locus as the most critical among COVID-19-related regions, which has been identified and highlighted by previous GWASs and functional analyses (41–43).

Our study revealed loci within the 9q34.2 and the 12q24.13 region as influencing both asthma and COVID-19 hospitalization. The *ABO* gene is the single gene within the 9q34.2 locus. Previously GWASs have identified it as a risk gene for critical illness of COVID-19 (41, 42) and asthma (26), while the present study suggests its involvement of COVID-19 infection and hospitalization as well. In previous studies, blood group A was associated with an increased risk for severe COVID-19 (OR = 1.45), while blood group O was shown to confer a protective effect (OR = 0.65) (42). Epidemiological studies reported a similar risk pattern for contracting COVID-19 (44, 45). Interestingly, *ABO* was also highlighted as a genome-wide gene for asthma by Han et al. (26), with blood group O being specifically reported as a risk factor for asthma in a recent review (46). Therefore, the effects incurred by the blood types on both diseases seem genuine.

Chromosome 12q24.13 contains a cluster of genes for the oligoadenylate synthase family (*OAS1, OAS2*, and *OAS3*). These enzymes synthesize 2’,5’-oligoadenylates (2-5As), which aid in degrading viral RNAs and inhibiting viral replication by activating latent RNase L (47). The association of *OAS2* with asthma was reported in an earlier GWAS (48). Moreover, one study showed that expression levels of OAS2 correlate with reticular basement membrane thickness (49). Notably, OAS2 was recently suggested as one of the hub genes for coordinating innate immune responses in COVID-19 and a potential to-be-augmented target for the treatments of this illness (50). In particular, inhibitors of endogenous phosphodiesterase 12 (PDE-12) enzyme, which cleaves the host 2-5As, were proposed for this purpose (51).

Our meta-analysis supports that the effects of variation within the *ABO* and *ATXN2* genes are shared between COVID-19 and asthma. Since *ATXN2* has been associated with asthma at the genome-wide level, our meta-analysis suggests *ATXN2* may be a novel risk gene for COVID-19. Ataxin-2, which is encoded by the *ATXN2* gene, is a multifunctional protein of the rough endoplasmic reticulum and plasma membrane (52), where it modulates mTOR signals by participating in its translational regulation by associating with polyribosomes (53). In stressed cells, ataxin-2 also is involved in the formation of stress granules, where untranslated mRNAs are translationally inhibited (54, 55). Notably, stress granules attract certain viral proteins, including that of positive-strand RNA viruses SARS-CoV-2 (56) and Zika (57). In fact, induced disassembly of the stress granules is required for the production of viral particles (58). While the role of ataxin-2 in supporting the replication of SARS-CoV-2 is yet to be investigated, Zika (ZIKV) decreases its viral production in response to ataxin-2 depletion (57). Moreover, the N protein of SARS-CoV-2 (56, 59) and ataxin-2 (60) both aid in the formation of high-density protein/RNA condensates through their intrinsically disordered regions, possibly competing with each other.

The role of ataxin-2 in immunity is less clear. Previous studies have connected the genetic variation in the *SH2B3/ATXN2* region with CD4+ T cells counts (61), and a variety of autoimmune conditions, including alopecia areata (62) and sarcoidosis (63).

Functional analyses showed that the set of 19 genome-wide risk genes for COVID-19 is expressed at a high level in the spleen, lung, and blood, supporting the involvement of the local immune responses in course of the COVID-19. Interestingly, GWAS hits of asthma were also enriched in three tissues mentioned above, and in the small intestine. Pathway analysis supports that the severity-related set of 19 genes predominately participates in cytokine and chemokine signaling, consistent with their enrichment in gene sets associated with several immune-related conditions. Our results strengthen the proposed viewpoint that COVID-19 progression depends on over-activated innate immunity and resultant ‘cytokine storm’.

The strengths of this study include the MR design, which is known to help avoid the causality pitfalls of traditional observational epidemiological studies. All or the vast majority of the participants were of European ancestry, reducing the potential population heterogeneity. Several limitations should be acknowledged, including pleiotropy as a potential source of bias capable of undermining the validity of an MR study. In the present study, both COVID-19 and asthma datasets contained samples from the UK biobank; this sample overlap may contribute to pleiotropy. However, the pleiotropy test revealed no indication of directional pleiotropy in the MR analysis.

In conclusion, our results suggest that genetic liability to asthma is associated with decreased risk for COVID-19 infection and hospitalization. This phenomenon may be due to the protective effects of ongoing inflammatory responses against the early stages of COVID-19.

## Data Availability Statement

Publicly available datasets were analyzed in this study. This data can be found here: COVID-19 Host Genetic Initiative (https://www.covid19hg.org/results/r4/) and The NHGRI-EBI Catalog (https://www.ebi.ac.uk/gwas/).

## Author Contributions

FZ contributed to the study design and data analysis. FZ and AB wrote the manuscript. All authors contributed to revising the work and approved the final manuscript.

## Funding

This work was supported by the National Natural Science Foundation of China (81471364).

## Conflict of Interest

The authors declare that the research was conducted in the absence of any commercial or financial relationships that could be construed as a potential conflict of interest.

## Publisher’s Note

All claims expressed in this article are solely those of the authors and do not necessarily represent those of their affiliated organizations, or those of the publisher, the editors and the reviewers. Any product that may be evaluated in this article, or claim that may be made by its manufacturer, is not guaranteed or endorsed by the publisher.
