# Supplementary material for: Causal Association and Shared Genetics Between Asthma and COVID-19
Source: Front Immunol. 2022 Mar 21;13:705379. doi: 10.3389/fimmu.2022.705379 (PMC8977836; doi:10.3389/fimmu.2022.705379)
Supplement: Supplementary file 1 [file DataSheet_1.doc]

**Supplementary File**

1. Mendelian randomization analysis

2. Supplementary Figure 1. Tissue enrichment analyses

3. References

**1. Mendelian randomization analysis**

To infer credible causal associations between neuropsychiatric traits and the COVID-19 phenotype, we performed Mendelian randomization analysis using R package TwoSampleMR v0.5.5 (1). We estimated causal effects by three complementary methods—inverse variance weighted (IVW), weighted median, and MR-Egger. These methods have different assumptions about horizontal pleiotropy (2). The IVW method was used as our primary method, which assumes a intercept of zero and provides a consistent estimate of the causality by a fixed-effect meta-analysis. The weighted median model places more weights on precise instrumental variables, therefore the estimate remains consistent even when up to 50% of the instruments are invalid or weak (3). The MR-Egger method assumed that the pleiotropic effects are independent and performs a weighted linear regression of the outcome coefficient on the exposure coefficient (2). The MR-Egger and weighted median models are less statistically powerful, but more robust to horizontal pleiotropy. The intercept of MR-Egger regression was used to evaluate the average horizontal pleiotropy (2). SNPs at genome-wide significance (P < 5×10^–8^) were selected as instrumental variants and further pruned using clumping r^2^ cutoff of 0.01. The MR-Egger model was used to evaluate potential directional pleiotropy (2).

**Supplementary Figure 1. Tissue enrichment analyses.** A: Gene-based tissue enrichment analysis of the 19 genes of COVID-19 hospitalization, DEG: differentially expressed genes; B: SNP-based tissue enrichment analysis of the asthma GWAS result.


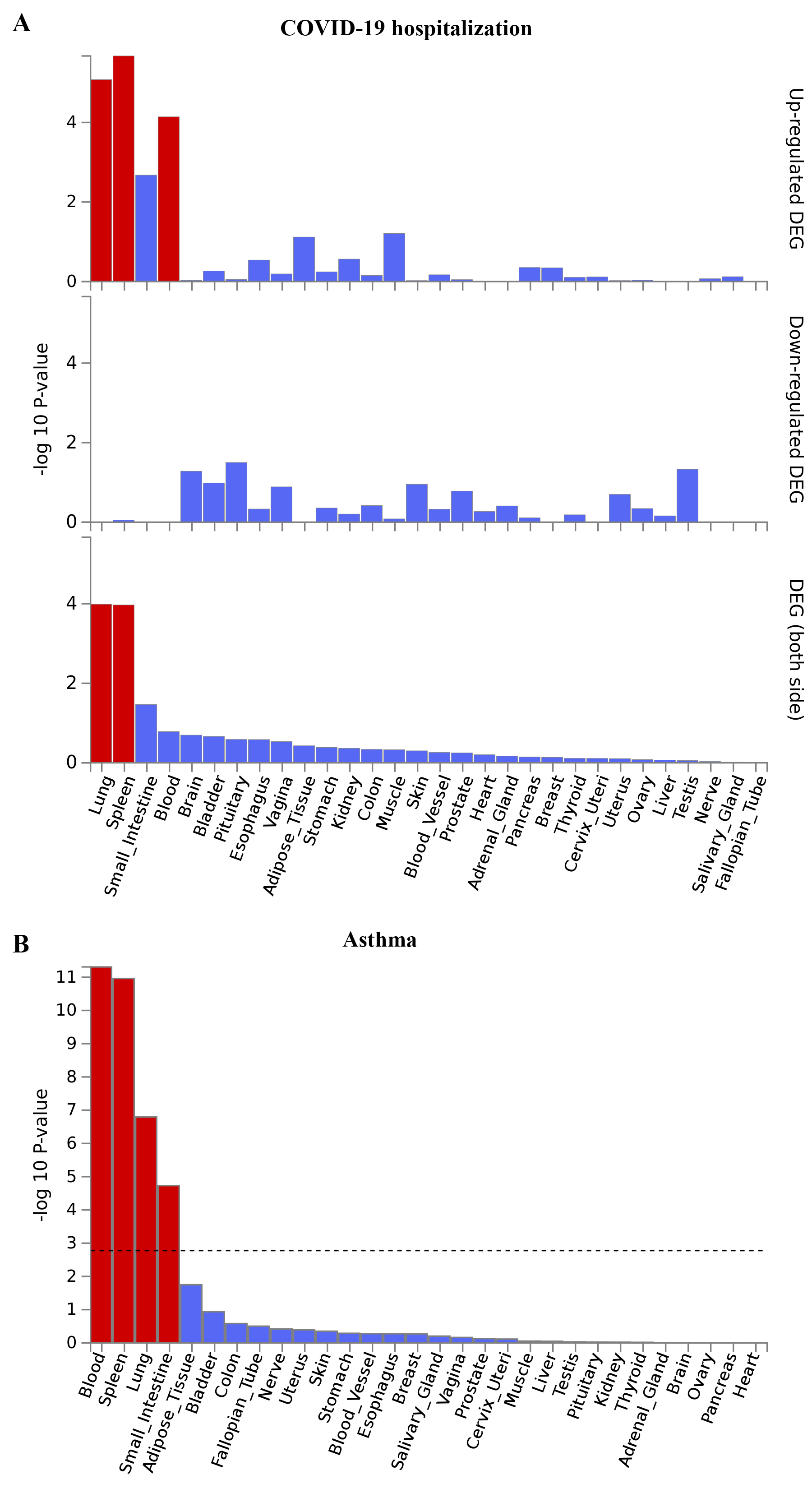


**3. References**

1. Hemani G, Zheng J, Elsworth B, Wade KH, Haberland V, Baird D, et al. The MR-Base platform supports systematic causal inference across the human phenome. *Elife* (2018) 7:e34408. doi: 10.7554/eLife.34408

2. Bowden J, Davey Smith G, Burgess S. Mendelian randomization with invalid instruments: effect estimation and bias detection through Egger regression. *Int J Epidemiol* (2015) 44(2):512-525. doi: 10.1093/ije/dyv080

3. Bowden J, Davey Smith G, Haycock PC, Burgess S. Consistent Estimation in Mendelian Randomization with Some Invalid Instruments Using a Weighted Median Estimator. *Genet Epidemiol* (2016) 40(4):304-314. doi: 10.1002/gepi.21965
